# Gendered Disparities in Quality of Cataract Surgery in a Marginalised Population in Pakistan: The Karachi Marine Fishing Communities Eye and General Health Survey

**DOI:** 10.1371/journal.pone.0131774

**Published:** 2015-07-17

**Authors:** Khabir Ahmad, Anthony B. Zwi, Daniel J. M. Tarantola, Abdul Qadeem Soomro, Rashid Baig, Syed Iqbal Azam

**Affiliations:** 1 School of Social Sciences, Faculty of Arts and Social Sciences, The University of New South Wales, Sydney, NSW, Australia; 2 Section of Ophthalmology, Department of Surgery, Aga Khan University, Karachi, Pakistan; 3 Office of Surgical Research, Department of Surgery, Aga Khan University, Karachi, Pakistan; 4 School of Public Health and Community Medicine, Faculty of Medicine, The University of New South Wales, Sydney, Australia; 5 Isra Postgraduate Institute of Ophthalmology, Al-Ibrahim Eye Hospital, Malir, Karachi, Pakistan; 6 Department of Community Health Sciences, Aga Khan University, Karachi, Pakistan; LV Prasad Eye Institute, INDIA

## Abstract

**Background:**

Marine fishing communities are among the most marginalised and hard-to-reach groups and have been largely neglected in health research. We examined the quality of cataract surgery and its determinants, with an emphasis on gender, in marine fishing communities in Karachi, Pakistan, using multiple indicators of performance.

**Methods and Findings:**

*The Karachi Marine Fishing Communities Eye and General Health Survey* was a door-to-door, cross-sectional study conducted between March 2009 and April 2010 in fishing communities living on 7 islands and in coastal areas in Keamari, Karachi, located on the Arabian Sea. A population-based sample of 638 adults, aged ≥ 50 years, was studied. A total of 145 eyes (of 97 persons) had undergone cataract surgery in this sample. Cataract surgical outcomes assessed included vision (presenting and best-corrected with a reduced logMAR chart), satisfaction with surgery, astigmatism, and pupil shape. Overall, 65.5% of the operated eyes had some form of visual loss (presenting visual acuity [PVA] < 6/12). 55.2%, 29.0%, and 15.9% of these had good, borderline, and poor visual outcomes based on presenting vision; with best correction, these values were: 68.3 %, 18.6%, and 13.1%, respectively. Of 7 covariates evaluated in the multivariable generalized estimating equations (GEE) analyses, gender was the only significant independent predictor of visual outcome. Women’s eyes were nearly 4.38 times more likely to have suboptimal visual outcome (PVA<6/18) compared with men’s eyes (adjusted odds ratio 4.38, 95% CI 1.96-9.79; *P*<0.001) after adjusting for the effect of household financial status. A higher proportion of women’s than men’s eyes had an irregular pupil (26.5% vs. 14.8%) or severe/very severe astigmatism (27.5% vs. 18.2%). However, these differences did not reach statistical significance. Overall, more than one fourth (44/144) of cataract surgeries resulted in dissatisfaction. The only significant predictor of satisfaction was visual outcome (*P* <0.001).

**Conclusions:**

The quality of cataract surgery in this marginalised population, especially among women, falls well below the WHO recommended standards. Gender disparities, in particular, deserve proactive attention in policy, service delivery, research and evaluation.

## Introduction

The most recent estimates by the World Health Organization (WHO) suggest that there were 285 million visually impaired people worldwide in 2010 [1]. Of these, 39 million people were blind and another 246 million had low vision. Cataract remains the leading cause of visual loss worldwide, accounting for nearly half of all blindness and a third of all visual impairment. Much of this burden occurs in low- and middle-income countries (LMICs) where access to eye care services remains uneven, with disadvantaged groups (notably women, elderly, rural and remote dwellers, the poor, and those with no school-based education) being disproportionately affected. Surgical removal of cataract remains the only effective treatment option. While substantial progress has been made in many LMICs to increase the number of cataract operations over the last 2 decades[2], quality of cataract surgery remains a relatively neglected aspect of eye care[3].

The WHO advises that more than 85% of cataract surgeries should achieve a good visual outcome (presenting visual acuity [PVA]: 6/18 or better) with fewer than 10% having borderline (<6/18-6/60), and less than 5% having poor (<6/60) outcomes. However, population-based data that exist suggest that a quarter to a third of all cataract surgeries in many LMICs leave people with poor visual outcome[4]. A common limitation of the published research is that it has focused almost exclusively on a single indicator (Snellen’s visual acuity) to measure the quality of cataract surgical care [5, 6], to the exclusion of other indicators including user perspectives of quality of care. In addition, these studies were largely conducted in general populations with insufficient focus on marginalised groups[7].

*The Karachi Marine Fishing Communities Eye and General Health Survey* [8, 9] was seminal in providing population-based data on eye disease burden, access to eye care, and eye health outcomes in a little-studied population of hard-to-reach fishing communities. We recently reported that 54.7% people aged ≥ 50 years in these communities had never had an eye examination, with perceived lack of need, financial hardships, and a range of “fears” and anxieties hindering access to eye care, despite a large unmet need [8]. This article reports our findings regarding the quality of cataract surgery and its association with gender and several other factors in that sample.

Pakistan has a sizable population of marine fishing communities, mostly living along its 1050 km Arabian Sea coastline. Fishing is a major source of income, employment, and food for these communities. Marine fishing today remains one of the most hazardous occupations in the world and most people who depend on this occupation live in abject poverty, suffering from government neglect and the uncertainties associated with weather and catch[10]. Worldwide, fishing communities have been largely neglected in health research.

## Materials and Methods

### Study design and setting

The methods of *The Karachi Marine Fishing Communities Eye and General Health Survey* have been described previously [8, 9]. Briefly, this was a population-based, cross-sectional survey, carried out from March 2009 to April 2010 in marine fishing communities in Keamari, one of the 18 towns in Karachi, Pakistan’s most populous city, located on the Arabian Sea coast. We purposely selected three islands, Baba, Bhit, and Shams Pir, and four coastal areas, Padar Ground, Kutchi Para, Babri Mosque, and Saddam Chowk in order to enable the study of the three major ethnic marine fishing communities residing in Karachi: Kutchi, Bengali, and Sindhi. These sites also exhibit very high rates of poverty and low levels of school-based education[8].

### Study participants and selection process

The study was focused on adults ≥ 50 years of age, given that worldwide two-thirds of all visual impairment occurs in this age group. Of 638 people who were included in this survey, 97 had undergone cataract surgery in one or both eyes and were included in the analysis.

Study procedures were explained to the eligible individuals and their informed verbal consent was obtained prior to participation. Written consent could not be taken because of the very low literacy rate in the selected population. An independent witness attested that the “Participant Information Statement had been read to and understood by” the study participant. Permission to conduct this research was obtained from the Federal Ministry of Health, Islamabad, Pakistan, and the City District Government, Karachi, Pakistan. Ethical approval for the research was obtained from the Human Research Ethics Committee of The University of New South Wales, Sydney, Australia (HREC 08181).

### Study variables

Socio-demographic and socio-economic variables including age, ethnicity, self-reported financial status of the household, and daily per capita income of the household have been previously defined[8, 9].

*Visual impairment* (VI), recorded for each eye and then for the better eye, was classified based on PVA as: no VI (≥ 6/12), mild VI (<6/12–6/18), moderate VI (<6/18–6/60), severe VI (<6/60–3/60) and blindness (<3/60 [11]). *Blindness* was further categorised as Blindness 1 (<3/60–1/60), Blindness 2 (<1/60–light perception), and Blindness 3 (No light perception). *Pupil shape*, assessed using direct ophthalmoscopy, was recorded as regular if it was round and irregular if otherwise. *Astigmatism* was defined as −0.5 D cylinder or more and grouped as: none (< -0.5 D cyl.), mild to moderate (-0.5 to -1.5 D cyl.), severe (> -1.5 to -3.5 D cyl.), and very severe (> -3.5 D cyl). Participants were asked if they were satisfied with their surgery. For those who had bilateral cataract surgery, satisfaction was assessed for each eye separately. *Uncorrected refractive error* was defined as PVA < 6/12 improving to 6/12 or better with best correction or pinhole. *Posterior capsule opacification* (PCO) was defined as the presence of a thickened posterior capsule in visual axis on slit-lamp examination, causing PVA <6/12. *Age-related macular degeneration* (ARMD) was diagnosed based on the presence of signs, such as drusen, retinal pigment epithelial changes, and subfoveal choroidal neovascularisation. *Glaucoma* was defined as evidence of glaucomatous damage to the optic nerve head with or without raised IOP (>21mmHg). *Optic neuropathy* was defined as the presence of disc swelling or pallor, or relative afferent pupillary defect. *Phthisis* was defined as a small shrunken globe due to trauma or severe infection. The cause was labeled as *amblyopia* if best-corrected visual acuity (BCVA) of <6/12 was not attributable directly to any underlying structural abnormality of the eye or visual pathways.

### Data collection process

A central survey workstation was established in each of the 7 survey sites where interviews and eye examination were held. Our survey team (and their roles) comprised a study coordinator (interviews and managing study team), supervisor (managing equipment and supplies), refractionist (visual acuity measurements), ophthalmologist (eye examinations), two local female workers (recruitment, translation where necessary, and assistance in vision testing), two local guides/social workers (community participation and household identification), and an ophthalmic technician (coordinating eye care).

Each subject underwent an interview, autorefraction, testing of PVA and BCVA, and an ophthalmic examination. Those who had self-reported diabetes or saw < 6/12 in either eye not attributable to cataract, refractive error or several other causes underwent dilated posterior segment examination. A reduced logarithm of the minimum angle of resolution (logMAR) chart was used to assess vision because it is considered more accurate than a Snellen’s chart [11]. Dilated posterior segment examination was performed using slit lamp and +90D lens. Intraocular pressure was measured using Goldmann applanation tonometer. All data were recorded on a survey instrument specifically designed for this survey.

### Study size

Sample size determinations were made using *Power Analysis and Sample Size* (PASS) software version 2008 (NCSS, Kaysville, UT, USA). At least 60 operated eyes in each group (i.e., men and women) were needed to detect a 25% difference in the rate of good visual outcome after cataract surgery between them (51.3% vs 26.3%), with 80% power at a significance level of 5% (two-sided). The hypothesis that women were less likely to have a good visual outcome (PVA ≥ 6/18) after cataract surgery than men was based on the findings of *Pakistan National Blindness and Visual Impairment Survey* [12]. The study found that 33.5% of the operated eyes among men had good visual outcome compared with 26.3% operated eyes among women.

### Statistical methods

Data were double entered in Microsoft Access (Microsoft Corporation, Redmond, WA, USA) database and analysed using Stata10.1 (Stata Corporation, College Station, TX, USA). The logMAR visual acuity scores were converted into standardised categories: no VI, mild VI, moderate VI, severe VI, and blindness. To allow for ease of comparison across studies, visual outcomes were further classified as good (PVA 6/6–6/18), borderline (<6/18–6/60), and poor (<6/60) according to WHO’s guidelines.

Simple frequencies and proportions were calculated to describe categorical variables. Gender differences in age at the time of surgery were compared using a two-sample independent t-test. Chi-square or Fisher’s exact test (2-tailed) was used to compare proportions (e.g., type of health facility in which cataract surgery had been performed, the rate of intraocular lens [IOL] surgery, and astigmatism) between men and women, and across other subgroups.

Generalized estimating equations (GEE) for binary outcomes were used to identify factors associated with 3 indicators of quality of cataract surgery: suboptimal visual outcome (PVA <6/18), dissatisfaction with cataract surgery, and irregular pupil. A substantial number of surgeries were bilateral (*n* = 48). GEE was used to overcome the problem of correlated data.

Covariates examined in relation to suboptimal visual outcome (PVA <6/18) were: current age (years), sex, ethnicity, self-reported financial status of the household, daily per capita income of the household, time since surgery, and IOL surgery. Covariates examined in relation to dissatisfaction were: age, sex, ethnicity, visual outcome of cataract surgery, self-reported financial status of the household, daily per capita income of the household, time since surgery, and IOL surgery. Covariates examined in relation to pupil shape were: current age, gender, ethnicity, self-reported financial status of the household, daily per capita income of the household, time since surgery, IOL surgery, and visual outcome. Covariates with *P* ≤ 0.2 in the bivariate analysis were evaluated in the multivariable analysis, statistical significance for which was set at *P* < 0.05.

## Results

Overall, 97 of 638 persons (15.2%) in *The Karachi Marine Fishing Communities Eye and General Health Survey* had undergone cataract surgery in one or both eyes. A higher proportion of women than men (17.3% or 56/324 vs. 13.1% or 41/314, *P* = 0.137) had undergone cataract surgery in one or both eyes. A significantly higher proportion of women’s than men’s eyes had had cataract surgery (13.4% or 87/648 vs. 9.2% or 58/628, *P* = 0.016). Of 145 cataract surgeries, 133 (91.7%) were with IOL implantation (Table 1). Women had a slightly lower rate of IOL surgery than men (89.7% vs. 94.8%). However, this difference was not statistically significant (*P* = 0.268). There were also no significant differences in the rate of IOL surgery by ethnicity and socioeconomic status (data not shown in the tables). Women underwent cataract surgery at a much younger age than men. Women had a statistically significantly lower mean age at surgery (59.0±12.2) than men (62.7±8.8), t (143) = 2.031, *P* = 0.044. There was no statistically significant differences in the type of eye care facilities (private, charitable or government) in which men and women had undergone cataract surgery (*P* = 0.478). None of the surgeries had taken place in an “eye camp” setting.

**Table 1:** Gender differences in intraocular lens surgery rate, visual outcome and dissatisfaction with surgery in *The Karachi Marine Fishing Communities Eye and General Health Survey* (*n* = 145 eyes).

Variable		Men’s eyes *n* = 58	Women’s eyes *n* = 87	All eyes *n* = 145
		Freq. (%)	Freq. (%)	Freq. (%)
**IOL Surgery**	Yes	55(94.8)	78(89.7)	133(91.7)
	No	3(5.2)	9(10.3)	12(8.3)
**Presenting vision** ^**a**^	No visual impairment ≥ 6/12	28(48.3)	22 (25.3)	50 (34.5)
	Mild visual impairment <6/12–6/18	15(25.9)	15 (17.2)	30 (20.7)
	Moderate visual impairment <6/18–6/60	10(17.2)	32 (36.8)	42 (29.0)
	Severe visual impairment <6/60–3/60	1 (1.7)	4 (4.6)	5 (3.4)
	Blindness 1 <3/60–1/60	0(0.0)	3 (3.4)	3 (2.1)
	Blindness 2 <1/60–Light Perception	2 (3.4)	8 (9.2)	10 (6.9)
	Blindness 3 No Light Perception	2 (3.4)	3 (3.4)	5 (3.4)
**Best-corrected vision**	No visual impairment ≥ 6/12	41(70.7)	41 (47.1)	82 (56.6)
	Mild visual impairment <6/12–6/18	6 (10.3)	11 (12.6)	17 (11.7)
	Moderate visual impairment <6/18–6/60	7 (12.1)	20 (23.0)	27 (18.6)
	Severe visual impairment <6/60–3/60	0(0.0)	4 (4.6)	4 (2.8)
	Blindness 1 <3/60–1/60	0(0.0)	2 (2.3)	2 (1.4)
	Blindness 2 <1/60–Light Perception	2 (3.4)	6 (6.9)	8 (5.5)
	Blindness 3 No Light Perception	2 (3.4)	3 (3.4)	5 (3.4)
**Dissatisfaction with surgery**	Yes	15(25.9)	25(29.1)	40(27.8)
	No	43(74.1)	61(70.9)	104(72.2)

^a^The WHO recommends that more than 85% of cataract surgeries achieve a good visual outcome (presenting VA of 6/18 or better) with fewer than 10% having borderline (PVA <6/18-6/60) and less than 5% having poor (<6/60) outcomes at presentation. With best correction, these values should be >90%, <5% and <5%, respectively

Results for each of the four outcome measures are reported below and in the Tables.

### Visual outcome of cataract surgery

Almost two-thirds (65.5%) of 145 operated eyes had some form of visual loss (PVA <6/12[Table 1]). Overall, 20.7%, 29.0% and 3.4% of the operated eyes had mild, moderate and severe visual impairment, respectively, while 12.4% eyes were blind. Among the 18 blind eyes, 5 had no light perception. When the data were classified according to WHO’s guidelines, 55.2%, 29.0% and 15.9% eyes had good, borderline and poor visual outcomes based on presenting vision, respectively (S1 Table). With best correction, these values were: 68.3%, 18.6%, and 13.1%. Visual outcomes were substantially different by gender, with a significantly higher proportion of women’s than men’s eyes having PVA of <6/12 (74.7% vs. 51.7%; *P* < 0.007) or PVA of <6/18 (57.5% vs. 25.9%; *P* < 0.001). These differences remained significant even after best correction. As shown in Table 2, three covariates—gender, self-reported financial status of the household, and IOL surgery—had a *P* ≤ 0.2 in the univariate GEE analysis of factors associated with suboptimal visual outcome (PVA < 6/18). In the final analysis, gender was the only significant independent predictor of visual outcome. Women’s eyes were 4.38 times more likely to have suboptimal visual outcome compared with men’s eyes (odds ratio 4.38, 95% CI 1.96–9.79; *P* < 0.001) after adjusting for the effect of self-reported financial status of the household. IOL surgery was not included in the final model because it did not have appreciable effect in the multivariable analysis.

**Table 2:** Uni- and multivariable GEE analyses of predictors of suboptimal visual outcome of cataract surgery (PVA < 6/18 [*n* = 145 eyes]).

Variable		*n*	VA<6/18 on presentationFreq (%)	Crude odds ratio(95% CI)	Adjusted odds ratio (95% CI)
**All**		145	65 (44.8)		
**Current age, years**	50–59	43	23 (53.5)	1.0	
	≥ 60	102	42 (41.2)	1.62 (0.74–3.54)	
	*P* value			0.224	
**Sex**	Male	58	15 (25.9)	1.0	1.0
	Female	87	50 (57.5)	4.02(1.84–8.80)	4.38 (1.96–9.79)
	*P* value			<0.001	<0.001
**Ethnicity** ^**a**^	Kutchi	113	51 (45.1)	1.08(0.46–2.56)	
	Non-Kutchi	32	14 (43.8)	1.0	
	*P* value			0.858	
**Self-reported financial status of the household** ^**b**^	“Fine”	22	6 (27.3)	1.0	1.0
	“Poor/Fragile”	123	59 (48.0)	2.45 (0.84–7.16)	3.01(0.98–9.27)
	*P* value			0.103	0.055
**Daily per capita income of the household, US dollars**	≤ 0.52	88	41 (46.6)	1.34 (0.64–2.81)	
	≥ 0.53	57	24 (42.1)	1.0	
	*P* value			0.432	
**Time since surgery**	< 4 years	68	27 (39.7)	1.0	
	≥ 4 years	77	38 (49.4)	1.55(0.77–3.15)	
	*P* value			0.217	
**IOL surgery**	Yes	133	57 (42.9)	1.0	
	No	12	8 (66.7)	2.80 (0.72–10.69)	
	*P* value			0.139	

^a^Non-Kutchis included Sindhis (*n* = 19 eyes), Bengalis (*n* = 10 eyes) and Other (*n* = 3 eyes).

^b^ Self-reported financial status of the household was examined by asking participants how their household financial status was. Responses to this open-ended question were grouped into the following categories: fine, “can just make both ends meet,” poor/weak, very poor/very weak, alternating between getting food and not getting food, unpredictable livelihood–sometimes you get it, sometimes you do not, no savings, need to work daily to earn enough to make a livelihood, and derives livelihood from charity/zakat. For the present analysis, these categories were further grouped as: “fine” and “poor/fragile.”

Of the 30 eyes with PVA of <6/12-6/18, 23 (76.7%) were the result of uncorrected refractive error, 3 (10%) posterior capsular opacification (PCO), 1 (3.3%) corneal scar, 1 (3.3%) amblyopia, and 2 (6.7%) age-related macular degeneration (S2 Table). Similarly, of the 65 eyes with PVA of <6/18 on presentation, 9 (13.8%) were the result of uncorrected refractive error, 26 (40%) PCO, 2 (3.1%) corneal scar, 4 (6.2%) phthisis, 3 (4.6%) high cylindrical error, 2 (3.1%) glaucoma, 1 (1.5%) surgery-related secondary glaucoma, 5 (7.7%) optic neuropathy, 7 (10.8%) age-related macular degeneration, 2 (3.1%) retinal detachment, 1 (1.5%) central retinal vein occlusion, 2 (3.1%) diabetic retinopathy, and 1 (1.5%) maculopathy. All four eyes with small shrunken globe and one eye with total corneal scar were attributable to endophthalmitis. Women’s eyes were 1.3 times more likely to have suboptimal visual outcome due to PCO than men’s eyes, but this difference was not statistically significant. Analysis of other causes by gender could not be undertaken because of inadequate cause-specific data.

### Dissatisfaction with cataract surgery

Overall, more than one fourth (44/144) of cataract surgeries resulted in dissatisfaction (Table 3). Understandably, those with poor or borderline visual outcomes (PVA < 6/18) were substantially more likely to be dissatisfied than those who had a good visual outcome (VA ≥ 6/18; 50.0% vs. 10.0%; *P* < 0.001). Variation in dissatisfaction rate by household financial status was marginally significant (*P* = 0.061) while that by current age (*P* = 0.802), gender (*P* = 0.570), ethnicity (*P* = 0.110), daily per capita income of the household (*P* = 0.890), time since surgery (*P* = 0.838), and IOL surgery (*P* = 0.632) were not significant. Variables evaluated in the multivariable GEE analysis were: visual outcome, self-reported financial status of the household, and ethnicity. Household financial status was not included in the final model because it had no appreciable effect. The final model showed that those with poor or borderline visual outcome compared with a good one were more likely to be dissatisfied with the outcome of cataract surgery (adjusted OR 9.71, 95% CI 3.92–24.03; *P*<0.001)—as were ethnic Kutchis compared with non-Kutchis (adjusted OR 2.99, 95% CI 0.91–9.84; *P* = 0.071).

**Table 3:** Uni- and multivariable GEE analyses of predictors of dissatisfaction with cataract surgery (*n* = 144 surgeries).

Variable		*n*	Not satisfiedFreq (%)	Crude odds ratio (95% CI)	Adjusted odds ratio (95% CI)
**All**		144	40(27.8)		
**Current age, years**	50–59	43	12 (27.9)	1.12 (0.48–2.62)	
	≥ 60	101	28 (27.7)	1.0	
	*P* value			0.802	
**Sex**	Male	58	15(25.9)	1.0	
	Female	86	25(29.1)	1.26 (0.56–2.84)	
	*P* value			0.570	
**Ethnicity**	Kutchi	112	35 (31.3)	2.48 (0.81–7.59)	2.99 (0.91–9.84)
	Non-Kutchi	32	5 (15.6)	1.0	1.0
	*P* value			0.110	0.071
**Outcome of cataract surgery**	Good ≥ 6/18	80	8 (10.0)	1.0	1.0
	Borderline or Poor <6/18	64	32 (50.0)	9.14 (3.75–22.26)	9.71 (3.92–24.03)
	*P* value			<0.001	<0.001
**Self-reported financial status of the household**	“Fine”	22	2 (9.1)	1.0	
	“Poor/Fragile"	122	38 (31.1)	4.56 (0.93–22.25)	
	*P* value			0.061	
**Daily per capita income of the household, US dollars**	≤ 0.52	87	24(27.6)	1.06(0.47–2.37)	
	≥ 0.53	57	16(28.1)	1.0	
	*P* value			0.890	
**Time since surgery**	< 4 years	68	20(29.4)	1.08 (0.50–2.35)	
	≥ 4 years	76	20(26.3)	1.0	
	*P* value			0.838	
**IOL surgery**	Yes	132	36(27.3)	1.0	
	No	12	4(33.3)	1.39(0.36–5.28)	
	*P* value			0.632	

### Pupil shape

As shown in Table 4, 137 operated eyes were included in this analysis. 6 eyes could not be included because they had corneal scar, corneal blood staining or phthisis bulbi while 2 eyes had missing data. Overall, 21.9% (30/137) eyes had irregular pupil. A disproportionately high percentage of eyes with post-operative PVA of <6/18 or with aphakia had irregular pupil. A higher proportion of women’s than men’s eyes had irregular pupil (26.5% vs. 14.8%). Of the 8 covariates examined in relation to the presence of irregular pupil in the univariate GEE analysis, post-operative PVA, sex, and IOL surgery had a *P* ≤0.2 and were then evaluated in the multivariable GEE model. After adjustment for sex, post-operative VA achieved borderline statistical significance (adjusted OR 2.36, 95% CI 1.00–5.59; *P* = 0.051). IOL surgery did not show any appreciable effect and was not retained in the final model.

**Table 4:** Prevalence of, and factors associated with, irregular pupil in the operated eyes (*n* = 137 eyes).

Variable		*n*	Irregular pupil Freq (%)	Crude odds ratio (95% CI)	Adjusted odds ratio (95% CI)
**Overall**		137	30 (21.9)		
**Age, years**	50–59	42	12 (28.6)	1.70(0.67–4.32)	
	≥ 60	95	18 (18.9)	1.0	
	*P* value			0.266	
**Sex**	Male	54	8 (14.8)	1.0	1.0
	Female	83	22 (26.5)	2.09 (0.79–5.50)	1.50(0.54–4.20)
	*P* value			0.137	0.441
**Ethnicity**	Kutchi	105	24 (22.9)	1.36 (0.44–4.14)	
	Non-Kutchi	32	6 (18.8)	1.0	
	*P* value			0.593	
**Self-reported financial status of the household**	“Fine”	22	4 (18.2)	1.0	
	“Poor/Fragile”	115	26 (22.6)	1.16 (0.34–4.02)	
	*P* value			0.814	
**Daily per capita income of thehousehold, US dollars**	≤ 0.52	83	17 (20.5)	1.0	
	≥ 0.53	54	13 (24.1)	1.17 (0.47–2.89)	
	*P* value			0.735	
**Time since surgery**	< 4 years	65	13 (20.0)	1.0	
	≥ 4 years	72	17 (23.6)	1.37 (0.57–3.28)	
	*P* value			0.482	
**IOL surgery**	Yes	127	26 (20.5)	1.0	
	No	10	4 (40.0)	2.76 (0.65–11.66)	
	*P* value			0.168	
**Presenting visual acuity (Post-operative)**	≥ 6/18	80	12(15.0)	1.0	1.0
	< 6/18	57	18(31.6)	2.63 (1.17–5.92)	2.36 (1.00–5.59)
	*P* value			0.019	0.051

### Refractive outcome

As shown in Table 5, astigmatism was evaluated in 113 operated eyes. 30 eyes did not undergo best-correction owing to media opacities or blindness while 2 eyes were of participants who were unable to attend the cluster examination center. Their visual acuity with pinhole, where obtainable, was considered as BCVA. Of 113 eyes, 48 (42.5%) had mild to moderate astigmatism while a quarter (23.9%) had severe or very severe astigmatism. When analysed by gender, a higher proportion of women’s eyes had severe or very severe astigmatism than men’s eyes (27.5% vs. 18.2%). However, this difference was not statistically significant (*P* = 0.258).

**Table 5:** Prevalence of astigmatism as assessed in 113 eyes that had undergone cataract surgery.

Degree of astigmatism (D cyl.)	Men’s eyes *n* = 44	Women’s eyes *n* = 69	All eyes *n* = 113
	Freq (%)	Freq(%)	Freq (%)
None (< -0.5)	15 (34.1)	23 (33.3)	38 (33.6)
Mild (-0.5)	3 (6.8)	8 (11.6)	11 (9.7)
Moderate (> -0.5 to -1.5)	18 (40.9)	19 (27.5)	37 (32.7)
Severe (> -1.5 to -3.5)	8 (18.2)	16 (23.2)	24 (21.2)
Very severe (> -3.5)	0 (0)	3 (4.3)	3 (2.7)

## Discussion

The right to health, enshrined in international law and many national constitutions and legislations, requires that health care interventions are of good quality[13]. Unfortunately, the quality of surgery in this marginalised population was of concern as two-thirds of 145 eyes that had undergone cataract surgery had some form of visual loss. 12.4% eyes were blind after operation. Women experienced substantially worse visual outcomes.

The WHO recommends that poor visual outcomes should be experienced in no more than 5% of eyes undergoing cataract surgery. The visual outcomes in our study are worse than the WHO recommended values but relatively better than those found in a number of other studies in LMICs[14]. Our study setting, Karachi, is an urban area with reasonably good facilities and with a number of active charity-based eye care organisations. Reasonably good access to skilled eye care professionals and IOL surgeries appears to be present; all surgeries in this population had been performed in static eye care facilities and 92% of these were with IOL. A suboptimal rate of IOL implantation and “eye camp” surgery have been found to be major contributors to poor visual outcomes in many resource-poor settings, including Pakistan [12].

The most striking finding in the present investigation was the poorer visual outcomes of cataract surgery among women than men. Women’s eyes compared with men’s eyes were 4.38 times more likely to have borderline or poor visual outcome (PVA<6/18). While a more meaningful comparison of our findings with existing literature is restricted by the limited number of studies that report gender-disaggregated visual outcome data, gender disparities of this magnitude have not been reported previously. Consistent with the findings of the *Pakistan National Blindness and Visual Impairment Survey* [12], women who manage to access cataract surgery do not benefit visually from it as much as they might. Our study found no statistically significant differences in the type of eye care facilities (private, charitable or government) in which men and women had undergone cataract surgery. However, their outcomes were worse, even if they did not voice their dissatisfaction as frequently as men. The question is whether women are getting the same quality of eye care as men. Are women receiving surgery from a subgroup of eye surgeons whose surgical skills and resources are inferior to those who treat men? Our data, by showing the presence of a relatively higher percentage of operated eyes with irregular pupil or astigmatism among women, may support this hypothesis, which needs to be investigated in future research in diverse health care settings in the country. The excess rate of poor visual outcome among women may partly explain why “fears” of operation or its poor outcomes, reported elsewhere, were far more prevalent among women with than men [8].

Globally, socioeconomic disparities in health status and quality of care are one of the most disturbing and challenging characteristics of health systems. In our study, there was a lack of a statistically significant association between socioeconomic status variables and visual outcome of cataract surgery. This could be due to the relatively homogeneous, low socioeconomic status of our study population, or the relatively small sample size that limited our statistical power to detect between-group differences.

More than two-thirds of the causes of suboptimal visual outcome identified in our study, such as refractive errors, PCO, severe infection and several other surgical complications are avoidable (preventable or treatable). Of particular concern is the high rate of endophthalmitis following cataract surgery (3.4%), which is generally reported to be less than 0.3%. Accurate IOL power calculation, effective infection control, and treatment of PCO should be a top priority of cataract surgical programmes in LMICs as should be upgrading the cataract surgery skills of many ophthalmologists and cataract surgeons [12, 15]. The reported rate of cataract surgery has increased in many LMICs, including Pakistan. Given the intense pressure to dramatically reduce the large surgical backlog and to extend cataract surgery to more people, care must be taken regarding the quality of these services and to ensure prospective monitoring to identify problems to be rectified.

In this population, one out of every four cataract surgeries was associated with dissatisfaction. Visual outcome was the only significant and independent predictor of satisfaction with surgery. Those with PVA < 6/18 were 10 times more likely to be dissatisfied with their surgery than those with VA ≥ 6/18. Satisfaction should be the most important quality indicator in cataract surgical care and a critical driver for the widespread uptake of cataract surgery in LMICs.

This research was carried out in hard-to-reach communities and was compounded by the unstable situation in Pakistan in general and Karachi in particular, with great difficulties associated with routine activity, including field research [16, 17]. This is one of the first population-based studies in an LMIC to employ a wide spectrum of indicators to assess quality of cataract surgery than mere visual outcome. Other strengths of this analysis include a relatively large, population-based sample of cataract surgeries given the hard-to-reach nature of this population, the detailed eye examination of participants (which involved the use of presenting and best corrected logMAR visual acuity testing, slit lamp biomicroscopy, and dilated posterior segment examination), the use of a higher visual acuity threshold than is traditionally used in population-based surveys, and attention to important potential confounders in the statistical analysis.

While our study does bring out an important finding of women having significantly poorer outcomes than men, the cross-sectional nature of our study and inadequate cause-specific data make it difficult to establish causality. It could not be determined whether some of the causes of suboptimal visual outcome preceded the surgery or vice-versa. Monitoring programmes or adequately sized prospective studies that take into account baseline differences in risk factors are needed to inform decision-making and practice. Another limitation of the study was differences in outcomes by ethnicity could not be meaningfully assessed because of a lower-than-expected rate of cataract surgery among ethnic Sindhis and Bengalis. One other report [9] from this study demonstrates a substantially lower uptake of eye care services by ethnic Bengalis compared with other ethnic groups. The literacy rate in this population was very low and the predictive utility of education also could not be examined. Larger sample sizes are required if inter-ethnic or other comparisons of quality, preferably also stratified by gender, are to be undertaken. Indeed, there is considerable need to undertake such studies given the direction of apparent disparities across the diverse population studied.

In summary, the quality of cataract surgery in this population, especially among women, falls short of the WHO recommended guidelines. The issue of poor quality of cataract surgery in LMICs has been highlighted by a number of previous studies and our work reinforces it. Quality of cataract surgery must receive at least as much attention as the quantity of surgery. This study recommends that, in LMICs, efforts and initiatives aimed to eliminate blindness and significant visual impairment due to cataract (the leading cause of visual loss) must focus, first and foremost, on improving the quality of existing cataract surgical services, especially among marginalised groups. Gender disparities, in particular, deserve proactive attention at policy and service response levels and in research and evaluation. With efforts to promote Universal Health Coverage receiving global attention in 2015 as the sustainable development goals are finalized, it is an opportune time to remind all of the need to reduce disparities and ensure equitable coverage of good quality services for all.

## Supporting Information

S1 Table Visual outcome of cataract surgery by selected characteristics (n = 145 eyes). (DOCX)

S2 Table Causes of suboptimal visual outcome of cataract surgery (n = 145 eyes). (DOCX)
