# Supplementary material for: Gendered Disparities in Quality of Cataract Surgery in a Marginalised Population in Pakistan: The Karachi Marine Fishing Communities Eye and General Health Survey
Source: PLoS One. 2015 Jul 17;10(7):e0131774. doi: 10.1371/journal.pone.0131774 (PMC4506126; doi:10.1371/journal.pone.0131774)
Supplement: S1 Table — (DOCX) [file pone.0131774.s001.docx]

**S1 Table. Visual outcome of cataract surgery by selected characteristics (*n*= 145 eyes).**

| **Variable** |  | *n* | **Visual outcome (based on presenting visual acuity)** | | | |
| --- | --- | --- | --- | --- | --- | --- |
|  |  |  | **Good** | | **Borderline** | **Poor** |
|  |  |  | ≥ 6/12 | < 6/12–6/18 | < 6/18–6/60 | < 6/60 |
|  |  |  | Freq (%) | Freq (%) | Freq (%) | Freq (%) |
| **All** |  | 145 | 50 (34.5) | 30 (20.7) | 42 (29.0) | 23 (15.9) |
| **Current age, years** | 50–59 | 43 | 15 (34.9) | 5 (11.6) | 15 (34.9) | 8 (18.6) |
|  | ≥ 60 | 102 | 35 (34.3) | 25 (24.5) | 27 (26.5) | 15 (14.7) |
| **Ethnicity** | Kutchi | 113 | 37 (32.7 | 25 (22.1) | 32 (28.3) | 19 (16.8) |
|  | Non-Kutchi* | 32 | 13 (40.6) | 5 (15.6) | 10 (31.3) | 4 (12.5) |
| **Education** | Any | 9 | 6 (66.7) | 1 (11.1) | 1 (11.1) | 1 (11.1) |
|  | None | 136 | 44 (32.4) | 29 (21.3) | 41 (30.1) | 22 (16.2) |
| **Self-reported financial status of the household** | “Fine” | 22 | 11 (50.0) | 5 (22.7) | 4 (18.2) | 2 (9.1) |
|  | “Poor/Fragile” | 123 | 39 (31.7) | 25 (20.3) | 38 (30.9) | 21 (17.1) |
| **Daily per capita income of the household, US dollars** | ≤ 0.52 | 88 | 26 (29.5) | 21 (23.9) | 31 (35.2) | 10 (11.3) |
|  | ≥ 0.53 | 57 | 24 (42.1) | 9 (15.8) | 11 (19.3) | 13 (22.8) |
| **Time since surgery** | < 4 years | 68 | 29 (42.6) | 12 (17.6) | 18 (26.5) | 9 (13.2) |
|  | ≥ 4 years | 77 | 21 (27.3) | 18 (23.4) | 24 (31.2) | 14 (18.2) |
| **IOL surgery** | Yes | 133 | 49 (36.8) | 27 (20.3) | 41 (30.8) | 16 (12.0) |
|  | No | 12 | 1 (8.3) | 3 (25.0) | 1 (8.3) | 7 (58.3) |

*Non-Kutchis included Sindhis (*n*=19 eyes), Bengalis (*n*=10 eyes) and Others (*n*=3 eyes).
