# Supplementary material for: Gendered Disparities in Quality of Cataract Surgery in a Marginalised Population in Pakistan: The Karachi Marine Fishing Communities Eye and General Health Survey
Source: PLoS One. 2015 Jul 17;10(7):e0131774. doi: 10.1371/journal.pone.0131774 (PMC4506126; doi:10.1371/journal.pone.0131774)
Supplement: S2 Table — (DOCX) [file pone.0131774.s002.docx]

**S2 Table. Causes of suboptimal visual outcome of cataract surgery (*n*= 145 eyes).**

| **Cause** | **Presenting VA <6/12-6/18** | | | | | | **Presenting VA <6/18** | | | | | |
| --- | --- | --- | --- | --- | --- | --- | --- | --- | --- | --- | --- | --- |
|  | **Male** | | **Female** | | **Total** | | **Male** | | **Female** | | **Total** | |
|  | Freq | % | Freq | % | Freq | % | Freq | % | Freq | % | Freq | % |
| Uncorrected refractive error | 11 | 73.3 | 12 | 80.0 | 23 | 76.7 | 2 | 13.3 | 7 | 14.0 | 9 | 13.8 |
| Posterior capsular opacification | 3 | 20.0 | 0 | 0 | 3 | 10.0 | 5 | 33.3 | 21 | 42.0 | 26 | 40.0 |
| Corneal scar | 0 | 0 | 1 | 6.7 | 1 | 3.3 | 2 | 13.3 | 0 | 0 | 2 | 3.1 |
| Pthysis | 0 | 0 | 0 | 0 | 0 | 0 | 2 | 13.3 | 2 | 4.0 | 4 | 6.2 |
| High cylindrical error | 0 | 0 | 0 | 0 | 0 | 0 | 0 | 0 | 3 | 6.0 | 3 | 4.6 |
| Amblyopia | 0 | 0 | 1 | 6.7 | 1 | 3.3 | 0 | 0 | 0 | 0 | 0 | 0 |
| Surgery-related secondary glaucoma | 0 | 0 | 0 | 0 | 0 | 0 | 0 | 0 | 1 | 2.0 | 1 | 1.5 |
| Glaucoma | 0 | 0 | 0 | 0 | 0 | 0 | 0 | 0 | 2 | 4.0 | 2 | 3.1 |
| Optic neuropathy | 0 | 0 | 0 | 0 | 0 | 0 | 0 | 0 | 5 | 10.0 | 5 | 7.7 |
| Age related macular degeneration | 1 | 6.7 | 1 | 6.7 | 2 | 6.7 | 3 | 20.0 | 4 | 8.0 | 7 | 10.8 |
| Retinal detachment | 0 | 0 | 0 | 0 | 0 | 0 | 0 | 0 | 2 | 4.0 | 2 | 3.1 |
| Central retinal vein occlusion | 0 | 0 | 0 | 0 | 0 | 0 | 1 | 6.7 | 0 | 0 | 1 | 1.5 |
| Diabetic retinopathy | 0 | 0 | 0 | 0 | 0 | 0 | 0 | 0 | 2 | 4.0 | 2 | 3.1 |
| Maculopathy | 0 | 0 | 0 | 0 | 0 | 0 | 0 | 0 | 1 | 2.0 | 1 | 1.5 |
| Total | 15 | 100 | 15 | 100 | 30 | 100 | 15 | 100 | 50 | 100 | 65 | 100 |
